# Taking the Translational Science Benefits Model from concept to operationalization: opportunities and challenges in defining impact using the Translational Science Benefits Model

**DOI:** 10.3389/fpubh.2025.1612590

**Published:** 2025-09-12

**Authors:** Jessica Sperling, Eman Ghanem, Stella Quenstedt, Tarun Saxena

**Affiliations:** ^1^Clinical and Translational Science Institute, Duke University School of Medicine, Durham, NC, United States; ^2^Social Science Research Institute, Duke University, Durham, NC, United States

**Keywords:** translational science, translational research, evaluation, scientific impact, research operations, Translational Science Benefits Model (TSBM)

## Abstract

The Translational Science Benefits Model (TSBM) was developed to conceptualize and communicate the benefits and impact of translational research. While the TSBM was developed as a conceptual model rather than an operational process, it can be integrated into operational processes to provide evidence and clearly explain the impact of translational research and translational science. This paper discusses the use of the TSBM not only as a conceptual framework but also as a program-integrated operational mechanism. First, it discusses three TSBM-informed programmatic processes for addressing intended and achieved impact: case studies, Pilots program reporting, and an organizational database. Then, it outlines the key factors emerging from these processes that should be considered before employing TSBM as an integrated structure for collecting information on translational research outcomes. In particular, this paper discusses key *who* questions with a focus on who codes or reports TSBM data, including accounting for the coder or reporter’s understanding of the TSBM, while balancing feasibility with validity. Key *how* questions including a specific focus on how potential TSBM outcomes are defined and determined. Key *when* questions address potential limitations or adaptation needs in TSBM-based measurement based on specific areas of focus, particularly workforce development and translational science-specific outcomes. Ultimately, this paper provides key lessons to consider when using the TSBM as a data collection tool and also explores opportunities to expand the utility of the TSBM as a data collection tool to understand, demonstrate, and augment the impact of translational research and science.

## 1 Introduction

The benefits of translational research (TR), which focuses on moving scientific discoveries from the laboratory settings to clinical applications for improved health outcomes, can be challenging to measure, as a TR enterprise intentionally spans across multiple domains. To address this and better communicate the positive effects and impacts of TR, various models and frameworks have been proposed. One frequently used model is the Translational Science Benefits Model (TSBM) (1, 2), which was developed to provide an organizing framework for conceptualizing and communicating the impact of TR.^1^ The TSBM, in its origin, focuses on 30 measurable benefits across four domains that span the following content areas: clinical benefits, community benefits, economic benefits, and policy benefits. Clinical benefits include factors such as biomedical technology and diagnostic procedures. Community benefits encompass improvements in healthcare delivery quality. Economic benefits include patents and licenses, as well as cost savings. Policy benefits include legislative or standards changes and the provision of expert testimony.

The TSBM has been a core impact framework used to address the outcomes of TR. Clinical and Translational Science Award (CTSA) Hubs, which are supported by the National Institute for Health’s (NIH) National Center for Advancing Translational Sciences (NCATS), are designed to support and advance translational research. They have used the TSBM as a central mechanism for conceptualizing, assessing, and communicating the impact of their work; it has served as a basis for research impact assessment and has provided a framing for public communication of impact across numerous Hubs (3–5). Beyond the CTSA utilization, TSBM utility has informed similar efforts in multiple other research entities and studies; for instance, the Centers for Diabetes Translation Research (CDTR) adapted the TSBM to develop its Research Impact Framework, and the QUARTET USA trial, a randomized study on hypertension treatment, utilized the TSBM to assess its impact across clinical and community domains (6, 7). The original TSBM publication by Luke et al. (1), has shown a clear scholarly impact with an upward trajectory in its citations over time. Citations more than doubled in recent years—from 15 in 2018–2019 and rising sharply to 36 in 2024–early 2025—indicating growing recognition and increasing integration of the TSBM framework into the broader TR or translational science (TS) discourse.

The originally-published TSBM set the expectation that future use could employ it as an assessment framework, but it was not designed as a specific data collection process or organizational operational structure. However, over time, TSBM has been increasingly used to more directly assess impact. It has supported evaluation of training programs, helping junior investigators articulate broader benefits of their work, and been used to map implementation project outcomes to specific TSBM domains (8, 9). In addition, many institutions have created TSBM-based case studies (5, 10), often as *ad hoc* efforts focused on select projects, but newer guidance and tools for developing impact profiles more efficiently could support broader organizational adoption (11). The embedding of the TSBM into research training and institutional evaluation systems demonstrates its practical utility, beyond theoretical application, as a base for advancing and measuring the impact of TR.

The transition from a conceptual framework to an operationalized system that is concretely used across programmatic activities introduces new questions and considerations about the model. In this article, we identify three active processes within the Duke Clinical and Translational Science Institute (CTSI), which has advanced translational research and translational science via NCATS CTSA support and other funding sources, that employ the TSBM as a mechanism for collecting and organizing data on benefits. We then outline key considerations emerging from these cases that are relevant to using the TSBM as a structure for data collection on outcomes, and which provide opportunities to consider modifications or enhancements to the model itself. We focus on four considerations: who is collecting data, how potential outcomes are conceptualized and applied in measurement, potential limitations of TSBM use for programs (as compared to research projects), and potential limitations of TSBM use for examining TS (as compared to TR) impact. This publication offers key insights for using the TSBM as a data collection tool and highlights opportunities to expand its utility to understand, demonstrate, and augment the impact of TR and TS.

## 2 Application of the Translational Science Benefits Model

**Figure 1:**
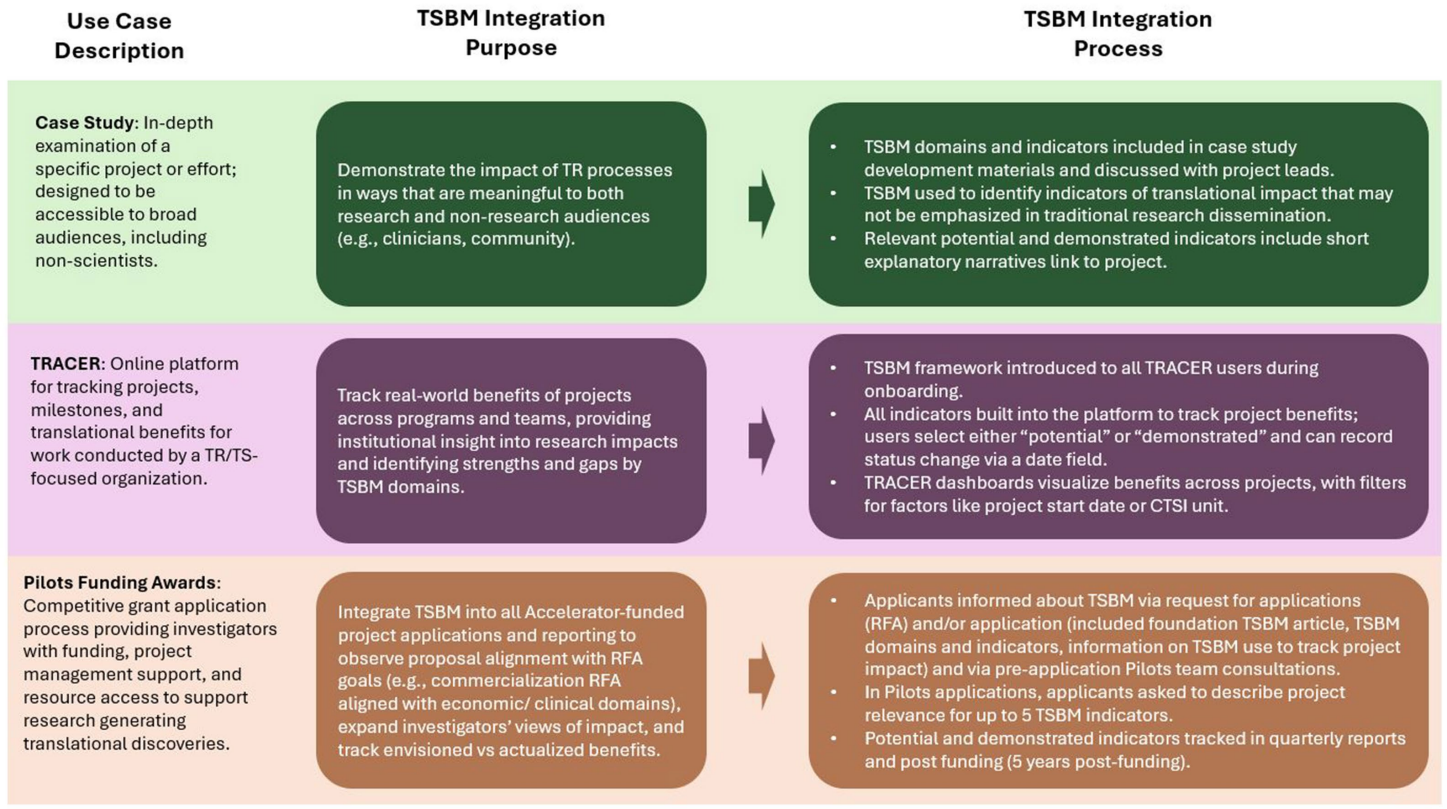
TSBM use cases overview.

Below, briefly in text and with additional information in Figure 1, we identify and describe three distinct opportunities to utilize the TSBM including in concrete operational efforts (hereafter described as “use cases”). These use cases were selected because they represent primary mechanisms the Duke CTSI has integrated TSBM into organizational processes; because they each differ in their purpose and the ways the TSBM was operationalized, thereby representing variation in forms of TSBM application; and because each use case raised specific considerations and questions regarding TSBM operationalization, providing a diverse basis for subsequent (Section 3) discussion of these points. These represent application at the level of cases (projects, specific initiatives), program, and an institutional enterprise.

### 2.1 Translational research/TSBM case study

A case study, or an in-depth examination of a specific subject (project), is a relatively common mechanism for applying the TSBM (5). As of early 2025, the authors’ research institution has published 7 case studies focused on a range of studies and programs that were supported by the CTSI by a variety of mechanisms (12). In a case study, the TSBM was operationalized using its domains to identify and prioritize specific indicators of translational impact that might not have been traditionally emphasized in research dissemination. For example, in a case study addressing research focused on maternal morbidity (13), the TSBM prompted a focus on the potential for guidelines that may result from the research, which would not have been captured using standard clinical metrics alone.

### 2.2 Pilot funding awards

The pilot awards program funds and supports a variety of projects that generate translational discoveries relevant to human health or disease. The CTSI has integrated the TSBM into all funded Pilots projects to track benefits over time, from applications to regular awardee updates. At the application stage, TSBM indicators were not specifically assessed as part of competitive proposal review; they were included to prompt investigator thinking about real-world impact from the project start and to provide a foundation for future tracking. Quarterly progress reports and annual follow-up reports incorporated the TSBM to track and update progress made towards benefits and any changes during the course of the project and for 5 years after the funding cycle.

### 2.3 Integration into organizational project and program tracking platforms

The TSBM has been integrated into an online relational data platform, the Translational Research Accomplishment Cataloguer (TRACER), which is used across the full Duke CTSI enterprise (14). It was developed at Duke and is utilized by Duke CTSI program staff and leadership; it houses information about all programs supported by the CTSI, and includes mechanisms for documenting and tracking milestones and benefits of projects supported by a CTSI. The TSBM was built into the data platform as specific fields available for projects in the database, providing an added mechanism of tracking real-world impact. The platform contains fields where teams can indicate which specific TSBM indicators are achieved or can potentially be achieved by each project (see Supplement for visual). This allows teams to trace the benefits for each individual project, and it permits a high-level view of real-world impact across an entire portfolio of projects.

## 3 Four considerations in operationalization of TSBM

The application of the TSBM across these specific use cases described above—TSBM case studies focused on individual projects, the pilot grants program, and the enterprise-level TRACER program to assess knowledge translation impact in the hub—helped to reveal key critical considerations across these operationalizations. Below, we highlight four considerations with implications for integrating the TSBM into program processes; these address questions about “who” (who determines benefits), “how” (how one determines potential benefits), and “when/how” (when and how the TSBM works to different applications).

### 3.1 Consideration # 1: Who determines relevant benefits?

While TSBM benefits include specific definitions, the individuals determining which benefits apply varies. For case studies, a bidirectional process between research teams and program staff and the Duke CTSI’s Evaluation, Improvement, and Impact team (EII) was used to determine relevant benefits. While research teams and program staff are not required to have any knowledge of the TSBM, members of EII have in-depth knowledge and experience with it. In this process, application of the TSBM is reviewed and/or discussed multiple times by both EII and research teams, to ensure agreement and proper application of the indicators. For TRACER, users are primarily program staff and researchers housed within Duke CTSI who have prior knowledge on the TSBM through use of the model in their own work and are provided TSBM materials to review prior to being onboarded to the TRACER platform. In addition, TSBM definitions and resources are built directly into TRACER where users would enter a project’s TSBM indicators. TRACER processes then rely on teams’ and their leadership’s assessments to determine relevant TSBM indicators for their projects. The EII team is available for additional guidance as requested but, based on the volume of projects as well as individual teams’ contextual knowledge, does not vet or inform each individual benefits selection. For pilot awards, relevant benefits are determined by the primary investigators applying for the award. These investigators are provided access to TSBM materials during their application, including Luke and colleagues’ foundational TSBM manuscript (1), the TSBM website, examples of case studies, TSBM definitions in the application form, and a pre-application discussion with the Pilots team during broader consultations; these are provided to create a TSBM foundation.

### 3.2 Consideration # 2: definitions of “potential” benefit

The concept of a *potential* benefit was a key feature of the TSBM in its early application in case studies. The process of translation can take many years to achieve the specific TSBM benefits. To address this, TSBM case studies often included a mechanism to document both demonstrated (benefits that had been achieved) and potential (benefits that had not yet been achieved but could be achieved by the project) indicators. While this is a key value of the TSBM, it also raised important questions about how *potential* can be defined in the operationalization process.

**Figure 2:**
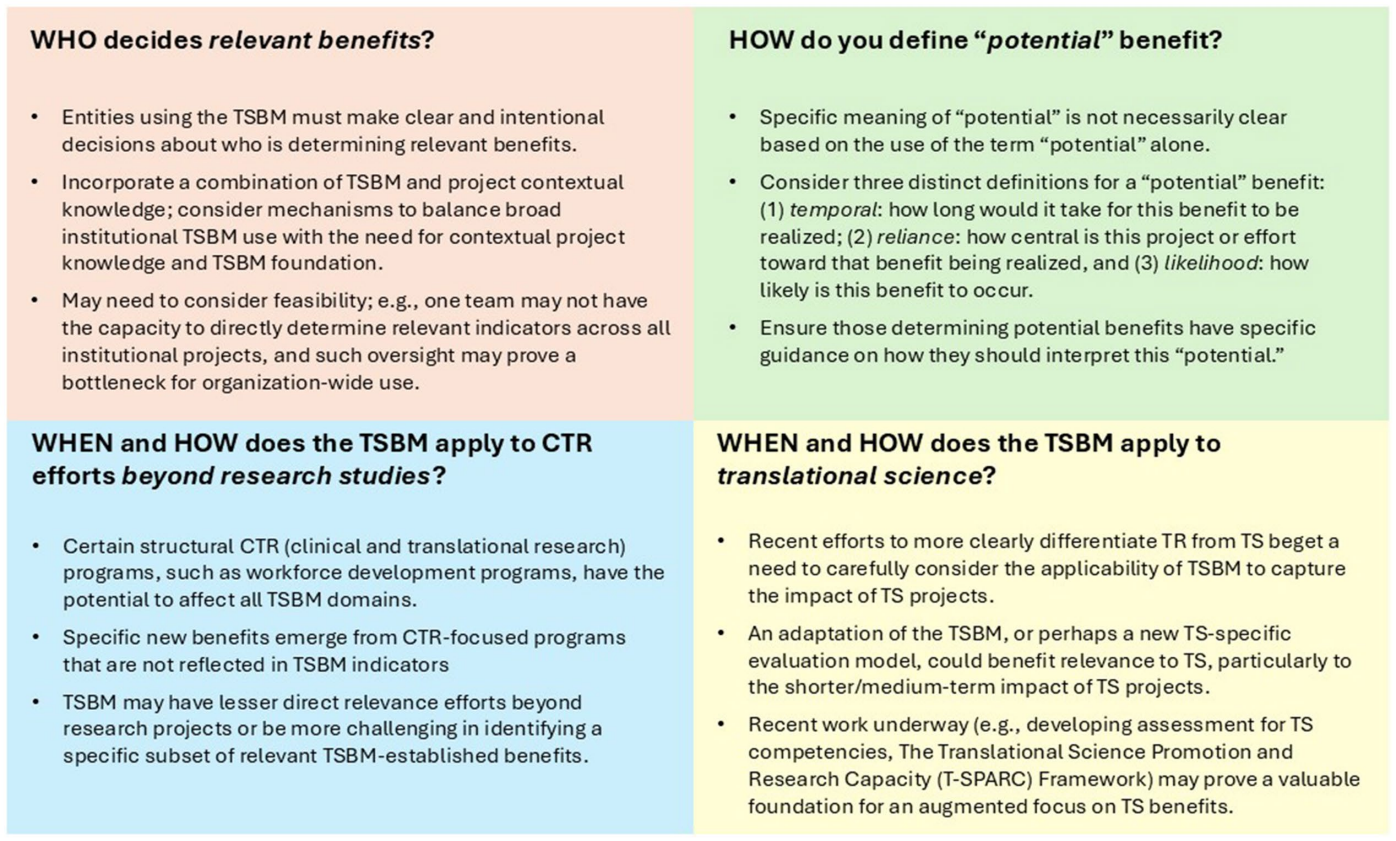
Lessons for application of TSBM in operational use.

In the case study application, potential was determined by the evaluation team and considered the anticipated timeframe for benefit realization, how central this project or effort is toward that benefit being realized, and the overall likelihood of a benefit occurring. In the TRACER database, the indication of a potential benefit was determined by the organizational team member who entered the project into the platform, with *ad hoc* review by CTSI leadership. In these cases, potential was defined by likelihood, as benefits that were expected with moderate to high confidence. In this context, potential was similarly defined by the perceived likelihood of a benefit occurring, where moderate to high confidence reflected a reasoned belief, based on organizational team members’ prior knowledge of and experience with similar projects, that the benefit was likely to emerge. In the pilot awards, a potential benefit and the definition of potential were determined by researchers. They were instructed to select expected benefits in their proposals, which were not explicitly defined but described as expected benefits aligned with the likelihood definition. See Figure 2 for a summary of ways to define “potential,” based on these applications.

### 3.3 Consideration # 3: application to research projects vs. capacity-development programs

TSBM use for case studies raised questions about its applicability to programs beyond research studies for which it was originally developed. Two specific case studies addressed clinical translational research workforce development programs rather than research projects. One case study focused on the development and implementation of North Carolina Central University’s Clinical Research Sciences program, which offers a certificate, minor, and bachelor’s degree (15, 16). Designed to build a highly trained workforce in clinical research and to increase access to entry into this workforce for all populations, the program aligned with many TSBM community-domain indicators, such as development of a *health education resource* and potential for improved *healthcare accessibility* through increased workforce representation. The team also proposed a new indicator under economic benefits: career access, given North Carolina’s rapidly growing clinical research industry. Overall, by advancing the number and breadth of individuals in a potential workforce and their competencies, the program could impact all TSBM domains. Another case study addressed YOJO (Your Journey), an online platform designed to facilitate persistence and sustainability in educational and professional development programs (17). Recognizing that pathway programs often operate in silos, YOJO was developed to connect them, simplify applications, track participants, and promote persistence among participants (18). When considering benefits, the team expanded beyond current TSBM benefits to add an additional potential benefit of workforce development, based on scholarship indicating that engaging individuals from all populations can strengthen the biomedical workforce (19–21). Like the Clinical Research Sciences program, YOJO has the potential to impact nearly all indicators via development of the clinical translational research workforce.

These case studies highlighted a challenge in applying the TSBM to capacity-building programs like education and training. Designed to build foundation for translational research, these programs often yield systemic, long-term impacts. Individual-level outcomes, such as skill development, knowledge gain, networking, and attitudinal change, are difficult to map to specific TSBM domains as one might with traditional research. In both case studies, the broad potential for impact across all TSBM domains revealed a limitation of the framework: it does not fully accommodate initiatives whose impact is foundational or systemic to enabling future translational activity. Additionally, the absence of categories such as “career access” and “workforce development” represents a meaningful gap when evaluating efforts to expand the translational science workforce. These insights point to the need to refine the TSBM to better capture the contributions of educational and workforce development programs to TR or TS ecosystem.

### 3.4 Consideration # 4: application to translational science

The TSBM was primarily designed to capture the outcomes of TR projects by focusing on benefits such as the development of new treatments, diagnostic tools, interventions, or community health change. In recent years, the National Center for Advancing Translational Sciences (NCATS) has implemented a strategic shift in its funding priorities, emphasizing TS as a priority, in addition to traditional TR, across CTSA hubs (22). As such, the NCATS required a shift to TS-focused Pilots projects and away from prior TR-focused projects. When considering how to advise applicants on TSBM benefits for their TS projects, the Pilots and EII teams questioned the fit. TR focuses on turning lab or clinical observations into health-improving interventions, often with focus on specific disease or patient population; TS focuses on scientific and operational principles underlying translational processes, with focus on addressing cross-cutting challenges across diseases or interventions to make translation more efficient across many diseases. The TSBM does not necessarily reflect the direct benefits of TS-specific projects and research. For instance, while some of TS developments may be captured within a TSBM benefit (e.g., new investigative procedures), other potential benefits such as improvements toward facilitating boundary-crossing collaborations or addressing persistent regulatory challenges, would not be as easily reflected in TSBM indicators.

## 4 Implications

This work provides evidence for methods to integrate the TSBM into operational processes within entities supporting TR. The TSBM can be applied in commonly used case studies, but it can also be used in other ways. For instance, it can be used in funding award processes to preemptively consider potential outcomes, to utilize the TSBM as a basis for regular reporting during the grant period, and to continue ongoing toward longer-term tracking of research outcomes. This provides a way to integrate the TSBM across all stages of a funded project and across a research portfolio. Additionally, it can serve as a part of organizational and program-level tracking in an entity that seeks to advance TR, helping to systematically evaluate impact across an organization and inform strategic decision-making. While the TSBM is valuable as a conceptual framework, its utility is further enhanced when applied in concrete, operational contexts.

The operational use of the TSBM, while valuable, also highlights certain challenges or considerations for the use of the TSBM. Although these may be viewed as limitations, they also present opportunities for refinement and further development of the framework. In our experience, key challenges include definitional inconsistencies and lack of specificity (e.g., as relevant to the “who” and “how” considerations), which can lead to measurement difficulties; this speaks to potential issues in data quality and validity and to the importance of data standards and operational guidelines to aid in transforming TSBM from theory to practice. With this basis, our applications of the TSBM highlight the need for clearer definitions, such as specifying what constitutes a *potential* benefit, and suggest the addition of new indicators within the framework, such as incorporating economic benefits related to workforce development programs. Additionally, the TSBM may not be fully suited for certain areas of the TR enterprise, such as training and workforce development programs, nor for assessing more direct TS outcomes. This limitation is especially relevant to TS/TR entities that are placing greater emphasis on advancing TS, as is the case for CTSAs based on emphasis in the most recent CTSA Funding Opportunity Announcement (22); while the TSBM remains relevant to ongoing TR efforts and even some TS components, it does not necessarily capture the full range of TS impacts. These limitations suggest the need for additional or complementary frameworks to more fully assess TS impact. For example, the NCATS TS principles (23) or the Translational Science Promotion and Research Capacity (T-SPARC) framework (24), which includes proximal and distal indicators of TS impact in a logic model format, can provide useful foundations for refining and expanding future TS impact frameworks. Figure 2 utilizes the four considerations to provide lessons in the application of the TSBM in operational use.

Such advancements of a framework or model based on application across new contexts is consistent with framework and model development more broadly; in other fields, applying conceptual models and frameworks in applied uses has been essential for refining their applicability and enhancing their impact in healthcare and medical research. For instance, within implementation science, the RE-AIM framework has developed and refined over time based on its use across public health, clinical, and community-based settings (25). Similarly, the Consolidated Framework for Implementation Research use identified the need for more explicit considerations of sustainability within the framework (26, 27). These adaptations have improved the framework’s ability to guide the design and evaluation of implementation strategies in various healthcare contexts. Similarly, very recently, the TSBM has begun to be adjusted or augmented. For instance, recent work expands the TSBM to formally include additional indicators, and additional research offers modifications for an implementation science application (9, 28, 29). Additional work has developed the model by integrating key tenets from engagement science (30), demonstrating processes for adapting the TSBM by directly linking it to other aligned conceptual work. Shifts to the TSBM, informed by our work, can guide additional future enhancements and modification opportunities.

Beyond development of the model itself, the work presented in this paper builds upon other emerging developments informing operational use of the TSBM. For example, recent efforts to develop and provide initial validation of an instrument assessing TSBM benefits have introduced greater specificity and clarity to key indicators, which could enhance consistency in implementation across different systems (31). This, combined with added specificity in defining what comprises a *potential* impact, could help to create greater validity and reliability to TSBM measurement and enhance potential for system-wide applications. Additional work on the TSBM, including studies featured in this special issue, represents important steps toward refining its applications, identifying limitations, and expanding its scope and utility. We recommend future work that continues to apply the TSBM in practical and operational contexts, both to maximize its impact in real-world settings and to identify ways in which the model can be continually improved and refined.

## Data Availability

The original contributions presented in the study are included in the article/Supplementary material. Further inquiries can be directed to the corresponding author.

## Footnotes

^1^The TSBM has been described as addressing translational research (TR) impacts and/or translational science (TS) impacts. Over time, TR and TS have come to be defined more distinctly (21). We discuss the TSBM in this section as most directly addressing TR impacts; this is further addressed later in this paper.
