# Supplementary figures and images for: Taking the Translational Science Benefits Model from concept to operationalization: opportunities and challenges in defining impact using the Translational Science Benefits Model
Source: Front Public Health. 2025 Sep 12;13:1612590. doi: 10.3389/fpubh.2025.1612590 (PMC12463953; doi:10.3389/fpubh.2025.1612590)

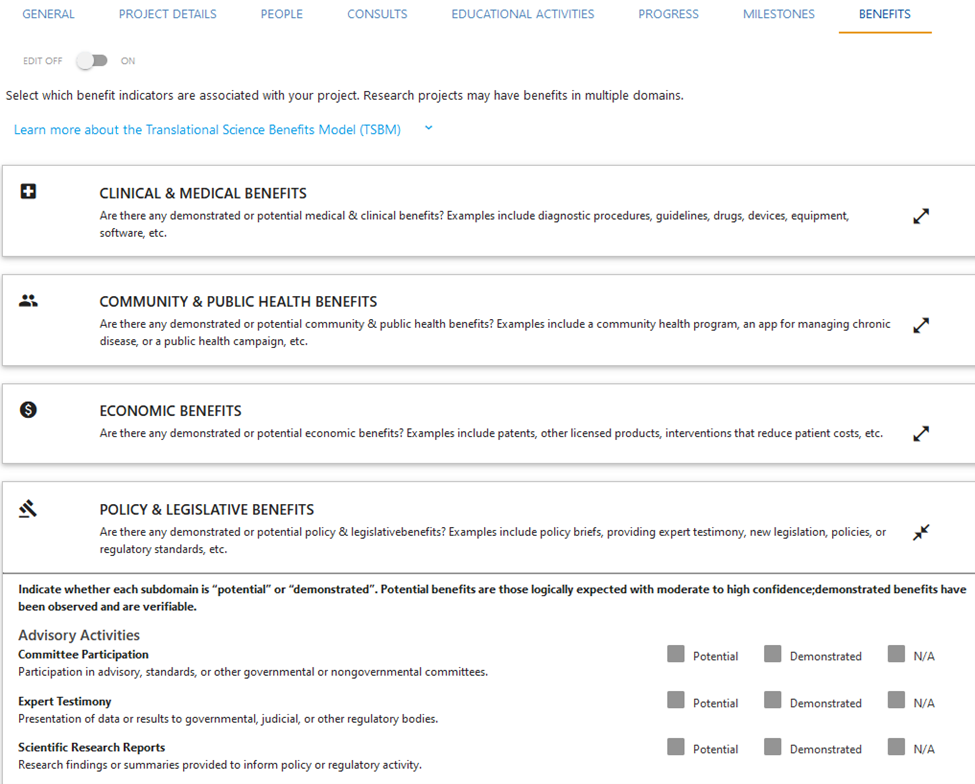

Supplement: Supplementary file 1 [file Image_1.TIF]
